# Binding of Nucleoid-Associated Protein Fis to DNA Is Regulated by DNA Breathing Dynamics

**DOI:** 10.1371/journal.pcbi.1002881

**Published:** 2013-01-17

**Authors:** Kristy Nowak-Lovato, Ludmil B. Alexandrov, Afsheen Banisadr, Amy L. Bauer, Alan R. Bishop, Anny Usheva, Fangping Mu, Elizabeth Hong-Geller, Kim Ø. Rasmussen, William S. Hlavacek, Boian S. Alexandrov

**Affiliations:** 1 Bioscience Division, Los Alamos National Laboratory, Los Alamos, New Mexico, United States of America; 2 Cancer Genome Project, Wellcome Trust Sanger Institute, Cambridge, United Kingdom; 3 X-Theoretical Design Division, Los Alamos National Laboratory, Los Alamos, New Mexico, United States of America; 4 Theoretical Division, Los Alamos National Laboratory, Los Alamos, New Mexico, United States of America; 5 Harvard Medical School, Beth Israel Deaconess Medical Center, Boston, Massachusetts, United States of America; Ecole Normale Supérieure, France

## Abstract

Physicochemical properties of DNA, such as shape, affect protein-DNA recognition. However, the properties of DNA that are most relevant for predicting the binding sites of particular transcription factors (TFs) or classes of TFs have yet to be fully understood. Here, using a model that accurately captures the melting behavior and breathing dynamics (spontaneous local openings of the double helix) of double-stranded DNA, we simulated the dynamics of known binding sites of the TF and nucleoid-associated protein Fis in *Escherichia coli*. Our study involves simulations of breathing dynamics, analysis of large published *in vitro* and genomic datasets, and targeted experimental tests of our predictions. Our simulation results and available *in vitro* binding data indicate a strong correlation between DNA breathing dynamics and Fis binding. Indeed, we can define an average DNA breathing profile that is characteristic of Fis binding sites. This profile is significantly enriched among the identified *in vivo E. coli* Fis binding sites. To test our understanding of how Fis binding is influenced by DNA breathing dynamics, we designed base-pair substitutions, mismatch, and methylation modifications of DNA regions that are known to interact (or not interact) with Fis. The goal in each case was to make the local DNA breathing dynamics either closer to or farther from the breathing profile characteristic of a strong Fis binding site. For the modified DNA segments, we found that Fis-DNA binding, as assessed by gel-shift assay, changed in accordance with our expectations. We conclude that Fis binding is associated with DNA breathing dynamics, which in turn may be regulated by various nucleotide modifications.

## Author Summary

Cellular transcription factors (TFs) are proteins that regulate gene expression, and thereby cellular activity and fate, by binding to specific DNA segments. The physicochemical determinants of protein-DNA binding specificity are not completely understood. Here, we report that the propensity of transient opening and re-closing of the double helix, resulting from thermal fluctuations, aka “DNA breathing” or “DNA bubbles,” can be associated with binding affinity in the case of Fis, a well-studied nucleoid-associated protein in *Escherichia coli.* We found that a particular breathing profile is characteristic of high-affinity Fis binding sites and that DNA fragments known to bind Fis *in vivo* are statistically enriched for this profile. Furthermore, we used simulations of DNA breathing dynamics to guide design of gel-shift experiments aimed at testing the idea that local breathing influences Fis binding. As a result, we show that via nucleotide modifications but without modifying nucleotides that directly contact Fis, we were able to transform a low-affinity Fis binding site into a high-affinity site and *vice versa*. The nucleotide modifications were designed only based on DNA breathing simulations. Our study suggests that strong Fis-DNA binding depends on DNA breathing - a novel physicochemical characteristic that could be used for prediction and rational design of TF binding sites.

## Introduction

Transcription factors (TFs), which play an important role in myriad cellular processes, are proteins that regulate gene expression through specific interactions with DNA at *cis* regulatory sites [1], [2], [3]. The binding sites in a genome of a given TF are generally only partially characterized. Importantly, although the modeling of TF binding sites is now being informed by high-throughput experimental mapping of TF binding sites, the detected binding sites are limited in precision [4]. The binding sites of a TF can be mapped on a genome-wide scale using various methods, such as chromatin immunoprecipitation followed by either microarray analysis (ChIP-chip) or sequencing (ChIP-seq) [5]. Another approach is protein binding microarray (PBM) analysis [6]. Application of these methods has revealed that the binding sites of a TF can be numerous, diverse, and difficult to represent with conventional models for TF binding sites [2], [7], [8], such as a consensus sequence or position weight matrix (PWM) model [9]. A single model may capture only a fraction of the binding sites of a TF detected via a high-throughput method, and, even with multiple models, it may be difficult to characterize the full spectrum of binding sites recognized by a TF [2], [10]. A possible explanation for these findings is that models neglect physicochemical features of DNA that are important for protein-DNA recognition.

A physicochemical feature of DNA that can affect protein-DNA recognition is the local sequence-specific structure [11]. Some TFs are known to recognize their binding sites predominantly through indirect readout, *i.e.*, recognition of DNA shape. Thus, by considering local DNA structure, one might expect that models for TF binding sites could be improved. Indeed, we recently reported that a method for predicting TF binding sites that makes use of local structural features of DNA, as well as other physicochemical features, outperforms PWM-based methods [12], [13]. Similar results have been reported by others [10], [14]. Although structure-based models of TF binding sites represent an advance in modeling, methods based on such models still fail to yield accurate predictions of binding sites for some TFs. For our method [12], [13], a well-characterized example is that of Fis, a nucleoid-associated protein and a transcription factor in *Escherichia coli* [15], [16]. Thus, it is unclear how best to take advantage of the physicochemical determinants of protein-DNA recognition, as is the exact nature of these determinants for specific TFs.

To better define the physicochemical features of DNA that contribute to protein-DNA recognition, we sought to determine whether known binding sites of Fis tend to share a sequence-dependent physicochemical feature that has not been considered in earlier work. Many physicochemical features of DNA are sequence dependent [17], [18]. As discussed above, one of these is the local DNA structure. Another is propensity for DNA breathing [19]. DNA breathing arises because DNA is subjected to thermal motion making it possible for the two strands of the double helix to locally and spontaneously open and re-close [19], [20]. Breathing dynamics as well as the melting behavior of short oligomers can be accurately predicted for many DNA sequences on the basis of the extended nonlinear Peyrard-Bishop-Dauxois (EPBD) mesoscopic model [21], [22], [23], [24]. DNA breathing dynamics can also be studied within the Poland-Scheraga [25] framework (e.g., see [26], [27], [28]). Variations in melting behavior [29], [30], [31], [32] as well as in breathing dynamics [33], [34], [35], [36] are believed to have functional consequences. DNA breathing dynamics that yield bubbles (*i.e.*, coherent relatively long-lived transient openings) are a characteristic feature of mammalian core promoters, see for example [37]. Sequences with a relatively high propensity to form bubbles have been found to overlap and affect transcription start sites [35], [38], [39], [40], replication origins [36], [41], correlate with TF binding sites and affect TF binding [42], [43], [44], [45], [46], [47], as well as to play role in formation of non-B-DNA structures [34] and in cytosine methylation [33]

A potential role for DNA breathing in Fis binding to DNA is consistent with known aspects of Fis-DNA interaction. Fis binding either requires or induces DNA bending with an angle of 50° to 90°, which can mediate translocation of superhelical energy to the promoter region, which in turn can activate transcription initiation [48], [49]. Hence, bending or flexibility is a potential necessary feature of a Fis binding site [50], [51]. The importance of bending has also been discussed in the literature [51]. Led by previously discovered correlations between DNA breathing and the *in vivo* binding of the mammalian transcription factor YY1 [47], we reasoned that breathing may also play a role in Fis binding. A study of Fis was also attractive because Fis-DNA interaction has been extensively studied [50], [51], [52], [53], [54], [55], [56], [57], [58]. For example, crystallographic and binding studies have identified rules that characterize the effects of sequence variations on the affinity of Fis-DNA binding *in vitro* [51], [58], [59].

Here, we report that Fis recognition of specific DNA sequences depends not only on direct points-of-contact with DNA, as elucidated in earlier work [51], [58], [59], but also on a particular DNA breathing profile. Based on our simulations and published *in vitro* binding data, we found a significant correlation between the affinity of Fis for a DNA sequence and the thermodynamic softness of that sequence, *i.e.*, the propensity for bubble formation. The computationally derived DNA breathing profile is characteristic of high-affinity *in vitro* Fis binding sites and is enriched to a statistically significant extent in genomic regions that associates with Fis according to ChIP-chip and ChIP-seq assays [56], [57], [60]. Finally, the genomic sequences carrying this breathing profile – characteristic for Fis binding sites *in vivo*, can be distinguished from ten times more randomly selected sequences from the *E. coli* genome on the basis of DNA breathing via supervised machine learning.

To experimentally test the influence of DNA breathing on Fis-DNA interaction, we used simulations of DNA breathing dynamics to guide modifications of well-characterized DNA sequences, as in earlier work [39], [46], [61]. We considered one sequence that binds Fis with relatively high affinity and one that binds Fis with relatively low affinity. We introduced base-pair substitution, mismatch, and O6-methylguanine modifications to alter the DNA breathing dynamics of these sequences while leaving nucleotides important for direct contact with Fis intact. The results of electrophoretic mobility shift assays (EMSAs) confirm that these modifications change affinity for Fis as predicted by our simulations.

## Results

### DNA breathing dynamics and Fis binding

**Figure 1:**
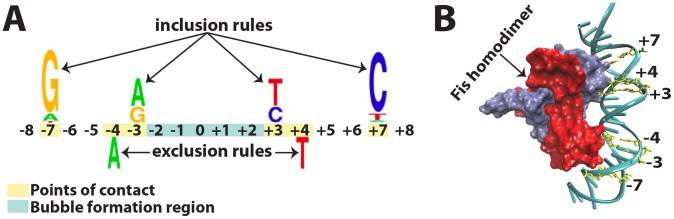
Fis-DNA points-of-contact and inclusion/exclusion rules. (A) Qualitative depiction of the sequence logo for a palindromic Fis binding site, emphasizing inclusion rules (above the numbers indicating the locations in the binding segment) and exclusion rules (below the numbers). The rules were derived from previous studies (see the main text). Yellow indicates direct points-of-contact or positions of inclusion/exclusion rules; blue indicates location of the Fis bubble formation region. The colors of the nucleic acids are chosen as the commonly used ones in consensus sequence logos. (B) Crystal structure example of Fis-DNA binding complex visualized by the data from PDB code 3IV5 as submitted in [51]. The nucleotides (direct points-of-contact) participating in the inclusion/exclusion rules are labeled and highlighted in yellow.

We sought to focus on DNA sequences that have the essential features of Fis binding sites. Previous studies, including systematic DNA base pair replacements in the Fis core-binding motif followed by a combination of EMSA and systematic evolution of ligands by exponential evolution (SELEX) experiments, have characterized the affinities of diverse Fis binding sites (Table S1) [50], [58]. We reviewed results of earlier studies of Fis binding [16], [50], [51], [53], [54], [57], [58] with the goal of gleaning clear patterns or rules that must be satisfied by a strong Fis binding site. These earlier studies taken together strongly suggest two inclusion-rules (requirements for nucleotides at specific positions as a condition for strong Fis binding), and one exclusion-rule (prohibition of a nucleotide at a specific position – the presence of such a nucleotide at a specific location obstructs Fis binding). These rules are illustrated in Figure 1A. Deviation from these rules hinders Fis binding [16], [50], [51]. In describing the rules of Figure 1, we use the IUPAC nucleic acid code and, without loss of generality, assume a palindromic motif (Figure 1A). Fis binding sites are usually, but not always [57], palindromic [58]. Thus, *in vitro* studies [50], [58] suggest that a high-affinity Fis binding site must contain either a guanine at the −7 position or a cytosine at the +7 position (*i.e.*, −7G or +7C is required). This condition is the first inclusion-rule. Similarly, −3R and +3Y are also common features of a high-affinity Fis binding site. These conditions are the second inclusion-rule. The exclusion-rule [51], [58] prohibits −4A or +4T, because adenine at the −4 position or thymine at the +4 position dramatically hinders binding. These three rules are consistent with (and partly derived from) recently determined Fis-DNA crystal structures [51], exemplified in Figure 1B. In both panels of Figure 1, direct Fis-DNA points-of-contact are highlighted in yellow.

**Figure 2:**
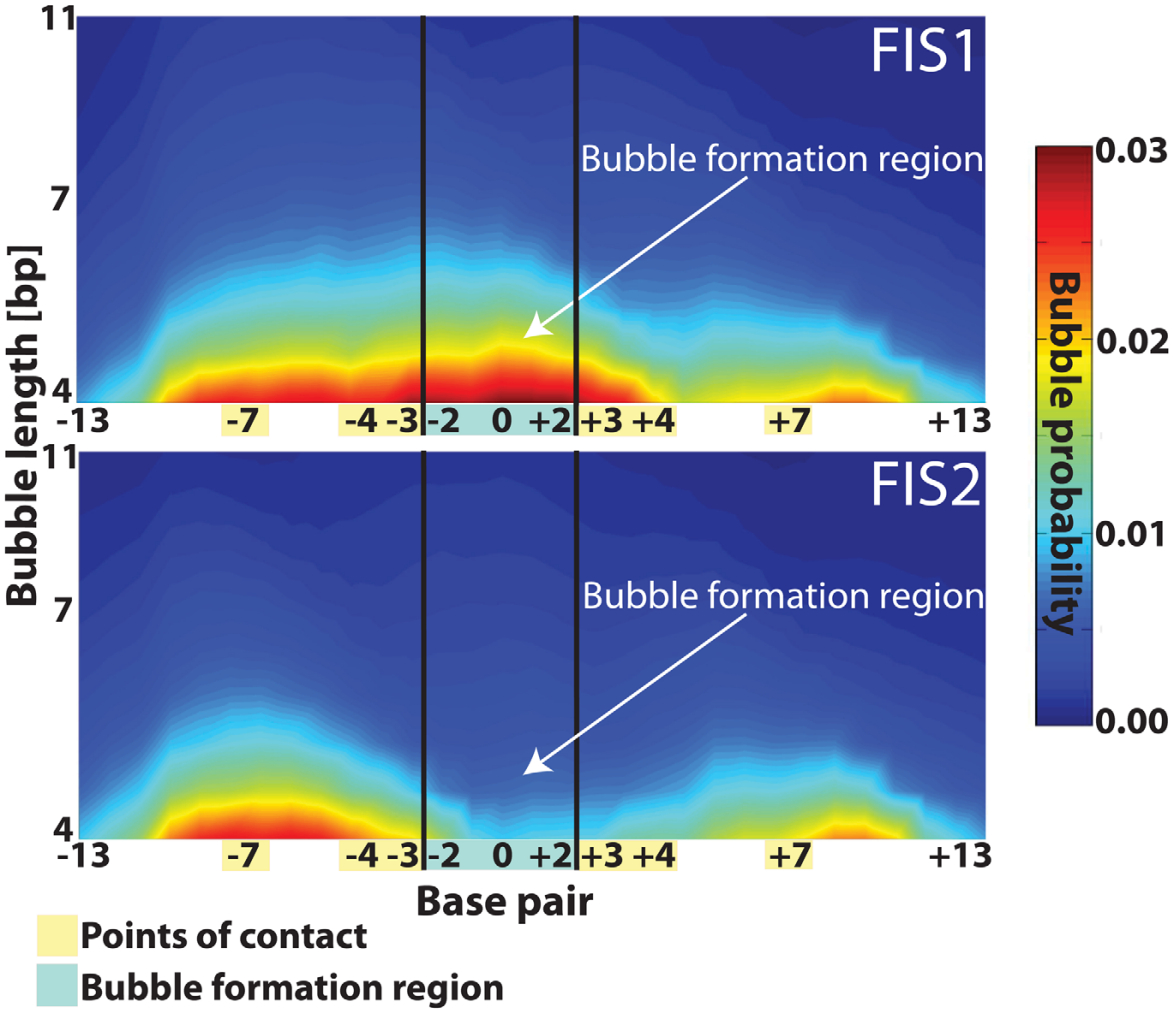
DNA local breathing dynamics of FIS1 and FIS2. The nucleotide positions are shown along the horizontal axis. Positions corresponding to points-of-contact are highlighted in yellow while the bubble formation region is highlighted in blue. The length of the transient bubbles (in number of base pairs [bp]) is shown along the vertical axis. The color map represents the probability for bubble openings where the red color denotes high probability and blue color denotes low probability. The name of the sequence for each variant is shown in the panel (all sequences can be found in Table 1).

To determine the local DNA breathing dynamics of sequences binding Fis differently (strongly or weakly) but satisfying the inclusion/exclusion rules for Fis binding sites and having the same direct points-of-contact, we performed simulations of EPBD Langevin dynamics [39]. Although EPBD does not include three-dimensional (3D) atomic coordinates and cannot be used to derive parameters that characterize local 3D-DNA structure (*e.g.*, roll, twist and tilt), which are considered in the SiteSleuth method [12], [13], the EPBD model provides a coarse-grained representation of DNA that accurately predicts DNA denaturation (melting temperatures of B-DNA oligomers) [23], [24], [62]. DNA breathing is connected with the propensity for local disruption of the hydrogen bonds between the complementary bases, and hence with DNA stability [63]. Importantly, we previously have demonstrated that our EPBD simulations can account for single-nucleotide polymorphisms responsible for non-local binding effects (in noncoding parts of the human genome) associated with schizophrenia [45], and that our simulations can serve to engineer DNA promoter functionality *in vitro* [39] and *in vivo* [61], to modify the strength of a TBP binding site (*i.e.*, the TATA-box) *in vitro* [46], and to predict the YY1 TF binding sites in cells [47]. To apply our computational framework to rationally design Fis binding sites, we initially considered two specific and well-characterized (as strong and weak) Fis binding sites without any changes. We simulated breathing dynamics of these sequences, which are called FIS1 and FIS2 (Table 1). These sequences have experimentally determined equilibrium dissociation constants (K_D_) for Fis binding and protein-DNA crystal structures. Both FIS1 and FIS2 satisfy all the inclusion/exclusion rules and have the same points-of-contact, but as shown in Figure 2, FIS1 and FIS2 have different breathing dynamics. These two sequences, each with 27 base pairs, differ only in five nucleotides in the middle of the 15 bp Fis core-binding region (Table 1). The difference between the two sequences is that a subsequence with high breathing propensity in FIS1, AATTT, is substituted with a subsequence with low breathing propensity in FIS2, GGCGC (Table 1). We will refer to the 5-nucleotide region that differs between FIS1 and FIS2 as the bubble formation region. This bubble formation region does not overlap with any of the Fis-DNA direct points-of-contact, but the breathing dynamics of this region affect FIS1 and FIS2 binding nonetheless. The reported K_D_ for Fis binding to FIS1 is 0.2 nM, whereas the reported K_D_ for Fis binding to FIS2 is 140 nM [51]. Replacement of the subsequence having high breathing propensity with the subsequence having low breathing propensity decreases the probability for local bubble formation in FIS2 more than 10-fold (Figure 2). This lower probability for bubble formation corresponds to a 700-fold lower affinity of Fis for FIS2 (*vs.* FIS1), which suggested that DNA breathing and Fis-DNA binding affinity might be correlated. To test this hypothesis, we performed simulations of breathing dynamics for an additional set of known Fis binding sites, 58 sites in total (Table S1). These sites have been characterized in previous *in vitro* Fis-DNA binding studies (see below).

**Table 1:** DNA oligomers investigated with EPBD Langevin dynamics.

	Position																											
Sequence Name	−13	−12	−11	−10	−9	−8	−7	−6	−5	−4	−3	−2	−1	0	+1	+2	+3	+4	+5	+6	+7	+8	+9	+10	+11	+12	+13	Modifications
	Left Flank						Core-binding Region															Right Flank						
**FIS1**	A	A	A	T	T	T	**G**	T	T	**T**	**G**	A	A	T	T	T	**T**	**G**	A	G	**C**	A	A	A	T	T	T	
**FIS1S**	c	g	c	T	g	T	**G**	c	g	**T**	**G**	A	A	T	T	T	**T**	**G**	c	G	**C**	A	c	g	c	T	g	**Substitutions (lower case)**
**FIS2**	A	A	A	T	T	T	**G**	T	T	**T**	**G**	G	G	C	G	C	**T**	**G**	A	G	**C**	A	A	A	T	T	T	^m^G = O6-methylguanine
**FIS2^m2^**	A	A	A	T	T	T	**G**	T	T	**T**	**G**	**^m^**G	G	C	**^m^**G	C	**T**	**G**	A	G	**C**	A	A	A	T	T	T	**two O6-methylguanine**
**FIS2^m3^**	A	A	A	T	T	T	**G**	T	T	**T**	**G**	**^m^**G	**^m^**G	C	**^m^**G	C	**T**	**G**	A	G	**C**	A	A	A	T	T	T	**three O6-methylguanine**
**FIS1**	A	A	A	T	T	T	**G**	T	T	**T**	**G**	*A*	*A*	*T*	*T*	*T*	**T**	**G**	A	G	**C**	A	A	A	T	T	T	
**|**	|	|	|	|	|	|	|	|	|	**|**	**|**						**|**	**|**	|	|	|	|	|	|	|	|	|	**Mismatches (italic)**
**FIS2**	T	T	T	A	A	A	**C**	A	A	**A**	**C**	*C*	*C*	*G*	*C*	*G*	**A**	**C**	T	C	**G**	T	T	T	A	A	A	

Bold capital letters indicate direct points-of-contact or positions of inclusion/exclusion rules, which are located at: **−7;−4;−3**, and **+7;+4+3** positions; the location of the Fis bubble formation region is between −2 and +2 positions; low case letters indicate base pair substitutions; italic capital letters indicate complementary strand mismatches; ^m^G represents O6-methylguanine.

### Fis binding affinity correlates with the equilibrium DNA opening profile

**Figure 3:**
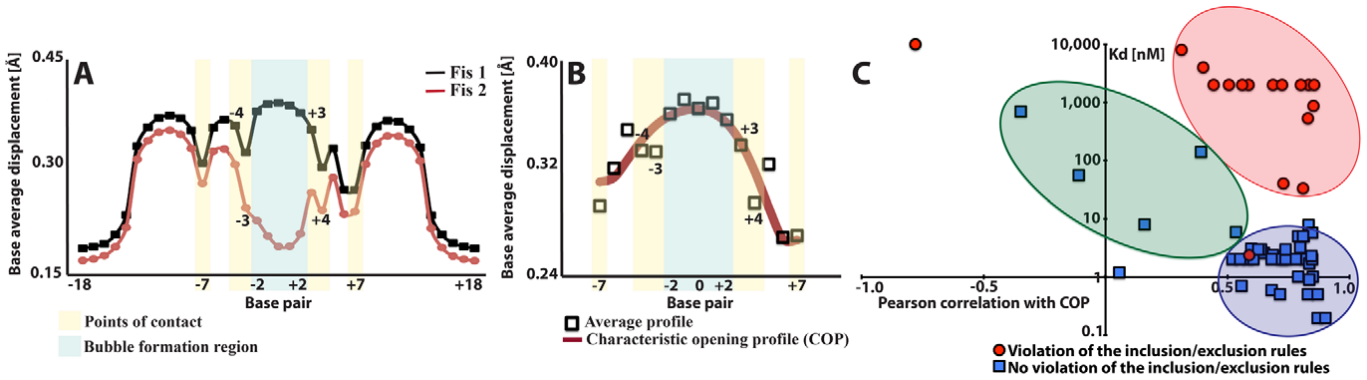
Correlation between Fis binding affinity and the generalized opening profile. (A) The MCMC average opening profiles (vertical axis in [Å]) of FIS1 and FIS2 as a function of the nucleotide position. The bubble formation region is highlighted in blue while the points-of-contact are highlighted in yellow. (B) Characteristic MCMC opening profile (COP) obtained as the average of the profiles (black squares) of the oligomers with in vitro Fis-DNA binding affinity with KD<1 nM (Table S1). The red line represents a polynomial fit to the data. The bubble formation region is highlighted in blue while the points-of-contact are highlighted in yellow. In panels (A) and (B), the horizontal axes indicate base pair position whereas the vertical axes indicate base pair average displacement (see Materials and Methods). (C) Schematic affinity shape-correlation diagram of the examined *in vitro* sequences. Each point represents an oligomer with specific direct points-of-contact, correlation with the COP, and measured dissociation constant KD (the data is from Table S1). The Pearson's correlation coefficient (horizontal axis) between the COP and the sequence shape and the KD (vertical logarithmic axis) are the (x, y) coordinates for each oligomer. The red circles depict sequences that violate at least two of the inclusion/exclusion rules while the blue squares correspond to the remaining sequences. The blue ellipse schematically depicts the majority of DNA sequences with good Fis-DNA binding (K_D_< = 3 nM), and the other two ellipses schematically depict the majority of sequences with low affinity to Fis caused by bad point-of-contacts (pink) or low correlation with COP (green).

Simulations of EPBD Langevin dynamics are computationally expensive. To more efficiently characterize DNA breathing for a large collection of sequences, we previously developed an efficient EPBD-based MCMC protocol that allows fast derivation of the DNA opening profile. The opening profile is based on calculations of the displacements/openings (see Materials and Methods) of each base pair in the DNA sequence from their equilibrium positions [23]. The base pairs openings are: i) connected at higher temperatures with DNA stability, as evidenced by the accuracy of DNA melting calculations [23]; ii) at physiological temperature and pH they effectively correspond to the flipping probability at various sites; and iii) they are free of the requirement for window averaging that is usually needed when thermodynamical or structural parameters of DNA are used to construct profiles characterizing regulatory elements [31], [64]. That is, the average opening profile represents a physical property of DNA at the single base pair level of resolution, connected to the temperature and pH [23], [39], [65]. Reanalyzing FIS1 and FIS2 with the EPBD MCMC protocol reveals that these two sequences exhibit different opening profiles in the bubble formation region (Figure 3A).

Previous *in vitro* studies have characterized Fis binding to a number of DNA oligomers. In Table S1, we summarize available binding data for 58 oligomers. We examined these sequences to determine whether the MCMC-derived DNA opening profiles can be used as a novel biophysical characteristic for describing Fis-DNA binding affinity. The MCMC profiles of the 10 sequences with K_D_≤1 nM, *i.e.*, with the strongest Fis binding (Table S1), were averaged, and a polynomial fitting function was used to capture the average profile of these high-affinity Fis binding sites. We will refer to this average profile as the characteristic opening profile (COP) of a strong Fis binding site (Figure 3B).

Analysis of the 58 oligomers revealed that the majority of sequences with strong affinity to Fis (K_D_< = 3.0 nM) do not violate the inclusion/exclusion rules and have individual average opening profiles that correlate with the COP, minimum Person correlation >0.51 (Figure 3C, the blue ellipse). The Pearson correlation coefficient is calculated as the correlation between the simulated residue displacements of a given sequence and the COP. The two exceptions are FIS16 and FIS40 (Table S1). The correlation cutoff of 0.51 can be compared to the correlations of the sequences used to derive the COP, which range from 0.56 (FIS37) to 0.9 (FIS12) (Table S1). Thus, the majority of previously *in vitro* characterized sequences, to which Fis binds strongly, are compliant with the inclusion/exclusion rules and have an opening profile that correlates with the COP. These results indicate that, at least for the available data, the MCMC-derived EPBD average opening profile of a sequence is associated to the affinity of Fis-DNA binding *in vitro*. We next decided to examine the interconnection between the COP and all experimentally known Fis binding sites in the *E. coli* K12 MG1655 genome.

### *E. coli* Fis binding sites are associated with a specific DNA breathing pattern

To compare the breathing dynamics of genomic regions containing Fis binding sites with the dynamics of randomly selected genomic regions, we collected a set of experimentally identified Fis binding sites (Tables S2 and S3). Recently, ChIP-chip and ChIP-seq assays have generated three independent high-throughput datasets [56], [57], [60] and a fourth set of Fis binding sites is available from a curated database [66]. Although there is some overlap at the gene level across the four datasets, there is almost no overlap at the binding-site sequence level [57], [60]. It has been suggested that this lack of overlap is due to limited sampling, meaning that the various studies may have each sampled only a distinct fraction of the Fis binding sites in the *E. coli* genome [60]. Below, we will examine the breathing dynamics of the Fis binding sites of Table S3. We will also examine the breathing dynamics of randomly selected sequences from the *E. coli* K12 MG1655 genome.

Table S3 lists a subset of the union of the four sets of experimentally identified Fis binding sites. Because the Fis core-binding sequence is 15 bp long (Figure 1), we filtered out experimentally identified Fis binding sequences shorter than 15 bp. We also filtered out sequences longer than 50 bp to focus only on high-resolution regions. Table S3 lists 1,449 Fis binding sites, which are more or less uniformly distributed across the *E. coli* genome. Of these 1,449 sequences, 1,411 are non-overlapping. Additionally, we randomly selected 14,110 non-overlapping 50 bp long sequences from the *E. coli* genome. These sequences were sampled from promoter, intergenic, and open reading frame regions in proportion to the frequency of the 1,411 Fis binding sites in each of these regions. In other words, 60% of the randomly selected sequences were drawn from promoter regions, 6% were drawn from intergenic regions (*i.e.*, regions between convergently transcribed genes), and 34% were drawn from open reading frames. This distribution of Fis binding sites across promoter, intergenic, and open reading frame regions is consistent with earlier observations [57].

**Figure 4:**
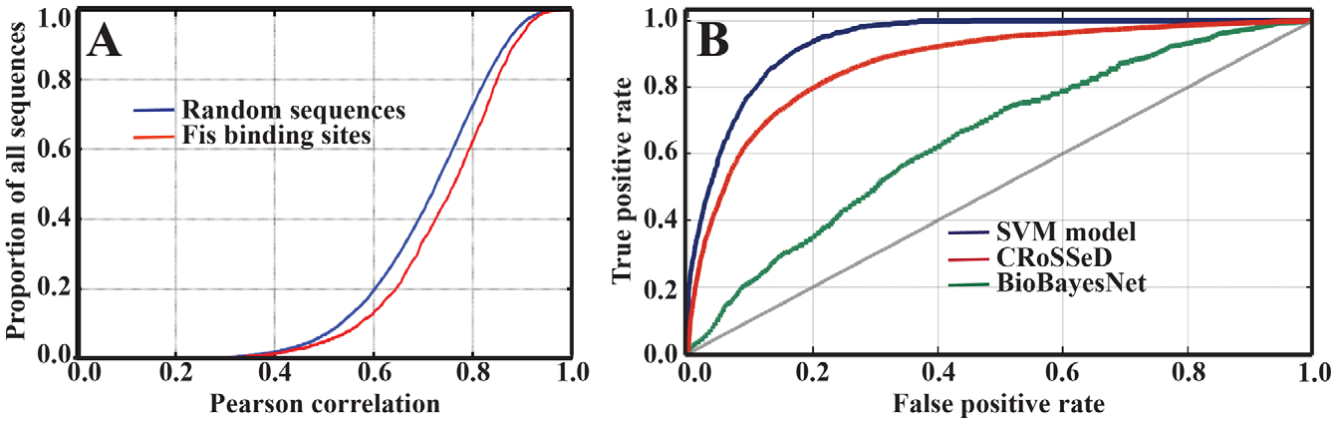
Fis i*n vivo* binding sites vs. randomly selected E. coli genomic sequences. (A) Cumulative density functions of subsequences with most significant correlation with COP. The Fis binding sites dataset is shown as a red curve while the random sequences dataset is depicted as a blue curve. The x-axis is the Pearson correlation coefficient while the y-axis is the proportion of subsequences with a maximum correlation with the COP profile worst or equal to the corresponding x value. (B) Receiver-operating curves base on our SVM model as well as models built with CRoSSeD and BioBayesNet. The x-axis depicts the false positive rate while the y-axis depicts true positive predictions. The blue curve corresponds to the results from our SVM classifier, the red curve to ones from the CRoSSeD model, and the green curve to ones from the BioBayesNet model.

We statistically compared the enrichment of the COP in the above two sets of sequences (Figure 4). For the sets of 1,411 known Fis binding regions and 14,110 randomly selected regions, we examined each sequence for the presence of 15 bp subsequences (the length of the Fis core-binding region) compliant with the exclusion rule. We considered these subsequences to be potential locations of Fis binding sites. The two inclusion rules were ignored here, because there are sequences that violate these rules but still bind Fis *in vitro*, although with relatively low affinity. Next, we calculated the Pearson correlation coefficient as above to characterize the similarity of the opening profile of each exclusion-rule compliant subsequence to the COP. Within a given genomic binding sequence, we first assumed that the subsequence with largest correlation coefficient is the *bona fide* Fis binding site. As shown in Figure 4A, known Fis binding regions are enriched for subsequences with opening profiles similar to the COP, relative to the set of randomly selected regions. The enrichment is statistically significant. With high confidence, we can say that the correlations with the COP for the two sets of sequences are drawn from different distributions: a two-sample T^2^ test yields a *p*-value of 2.69×10^−17^, and a two-sample Kolmogorov-Smirnov test yields a *p*-value of 6.64×10^−15^. To test this further, and without the assumption that the subsequence with largest correlation coefficient is the *bona fide* Fis binding site, we compared directly the distribution of the correlations to the COP of *all* 15 bp long subsequences at each genomic Fis binding set to the distribution of the correlations to the COP of *all* 15 bp long subsequences, at each 50 bp region in the random set. A two-sample T^2^ test yields a *p*-value of 4.17×10^−4^, and a two-sample Kolmogorov-Smirnov test yields a *p*-value of 5.56×10^−6^, thus further confirming that there is a statically significant enrichment of the COP profile in the set of known *in vivo* Fis binding sites.

Furthermore, we asked if the COP and the exclusion rule, alone, could be used to distinguish the *bona fide* Fis binding sites from ten times more background genome sequences. The results are shown in Figure 4B. As described in Materials and Methods, on the basis of the EPBD opening profiles, we trained a support vector machine (SVM) classifier to distinguish between the set of 1,411 presumed 15 bp *bone fide* Fis binding sequences and a set of 14,110 randomly selected 15 bp sequences. We evaluated the SVM classifier by comparing its receiver operating characteristic (ROC) curve with the ROC curves of two recently developed software tools for predicting TF binding sites, CRoSSeD [14] and BioBayesNet [67]. We selected these tools because they have been evaluated using an approach similar to the approach that we take here, *i.e.*, they have been shown to be superior to alternative methods at recognizing TF binding sites among a larger set of randomly selected sequences [14]. The ROC curves for our SVM classifier, CRoSSeD, and BioBayesNet are plotted in Figure 4B. As it can be seen, the performance of our SVM classifier is best. CRoSSeD and BioBayesNet implement general-purpose TF binding site prediction methods that incorporate DNA structural features, but these methods are not tailored specifically for prediction of Fis binding sites, so their relatively poor performance in the case of Fis binding is perhaps unsurprising. Although the model training is performed in a way that favors our SVM-based approach (*i.e.*, the SVM model is trained on DNA breathing profile that is enriched in the set of *bona fide* Fis binding sites), it is nevertheless encouraging that the SVM classifier performs best. Its superior performance indicates that the examined DNA breathing dynamics, which are associated with Fis binding sites *in vivo*, cannot be derived and recognized from the features considered in CRoSSeD and BioBayesNet, which include major/minor grove distance, width, and shape. From this result, it seems that DNA breathing is a novel binding characteristic and its incorporation as a novel feature into more sophisticated methods for predicting TF binding sites (*e.g.*, the SiteSleuth method [12], [13]), together with other important physicochemical features (*e.g.*, major/minor grove distance, width, and shape), would have the potential to improve prediction accuracy for genome-scale TF binding site prediction, at least for Fis binding sites. Such incorporation is not trivial and therefore it is left as a subject for a future study.

### Modification of the breathing and hence of the affinity of Fis binding sites

The available Fis-DNA crystal structures and the previous results of EMSA experiments have demonstrated that Fis contacts DNA only at a few intermolecular direct points-of-contact, which can significantly affect Fis binding affinity [51], [58], [59]. The existence of a correlation between the Fis binding affinity and the DNA breathing of the binding region, *in vitro*, as well as the enrichment of the COP among genomic Fis binding sites are indicative of a new biophysical characteristic of Fis binding. Namely, the bubble formation region should possess enhanced DNA local breathing dynamics. The enhanced DNA breathing dynamics of FIS1 and the suppressed breathing of FIS2 (associated with a 700-fold decrease in affinity) are consistent with this observation (Figure 2). Hence, we hypothesized that modifying Fis binding sites in accord with computationally predicted increasing or decreasing probability for local bubble formation would result, respectively, in stronger or weaker Fis-DNA binding.

#### Weakening Fis-DNA binding by suppressing breathing dynamics with base pair substitutions

**Figure 5:**
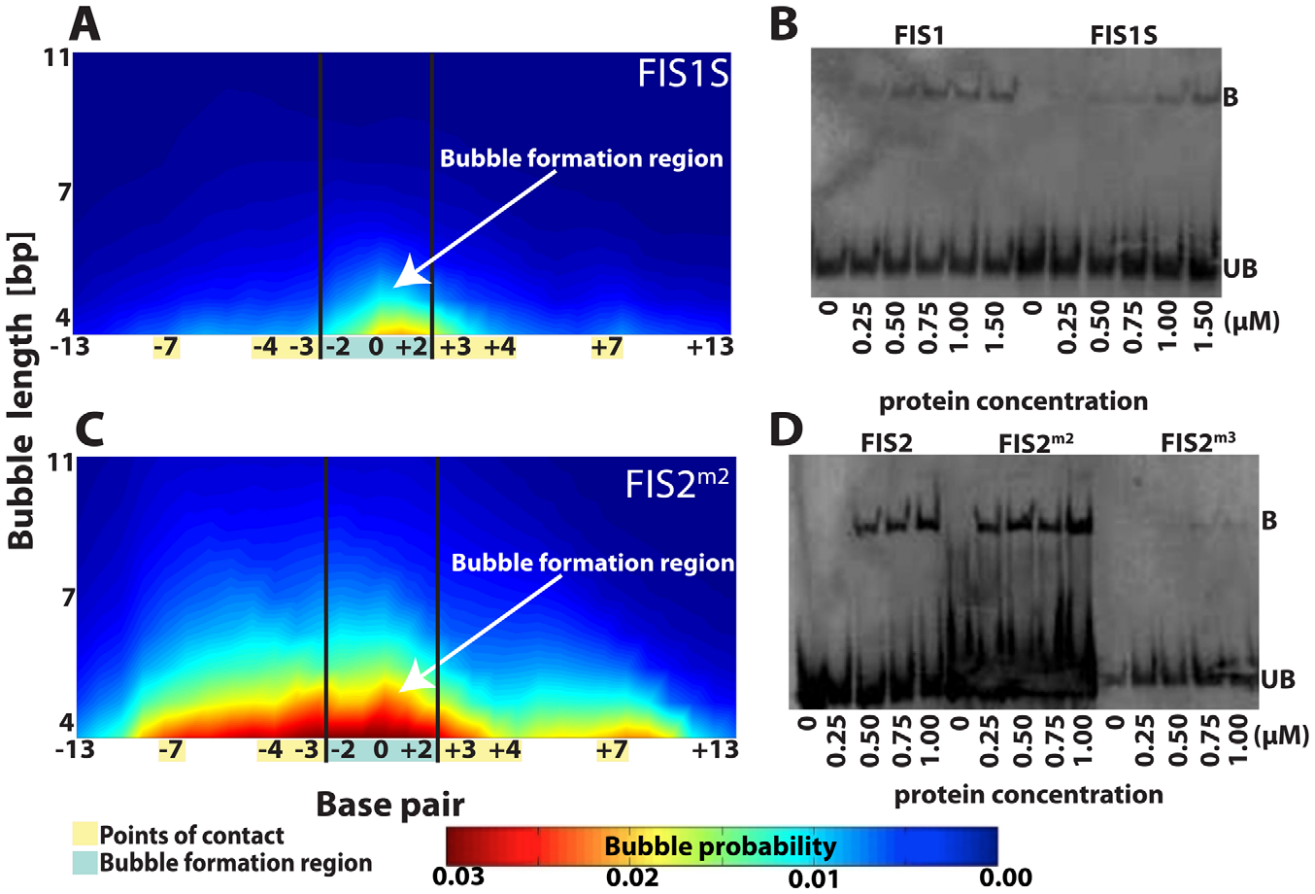
Fis binding site modifications, Langevin dynamics simulations of DNA breathing, and EMSA experiments, the first set. (A) Langevin dynamics simulations demonstrating suppressed local DNA breathing dynamics in the FIS1S sequence (compare to FIS1 in Figure 2). Base pair substitutions were made in the left and right flank regions of FIS1 to create a stiffer sequence while the direct points-of-contact remained unchanged (Table 1). (B) EMSA demonstrating the decrease in affinity of bound FIS1S sequence in complex with purified Fis protein vs. the FIS1 sequence. FIS1 and FIS1S oligonucleotides were constant at 100 nM, and Fis protein ranged from 0 to 1.5 µM. (C) Langevin dynamics simulations demonstrating enhanced FIS2m2 local DNA breathing dynamics (compare to FIS2 in Figure 2). FIS2m2 was designed by introducing two O6-methylguanine in the bubble formation region of FIS2 (Table 1) while the direct points-of-contacts remain unchanged. (D) EMSA demonstrating the increase in complex formation of FIS2m2 vs. FIS2 as well as the decrease in complex formation in FIS2^m3^ (third set of lanes). Oligonucleotide sequences were constant at 100 nM, and Fis protein ranged from 0 to 1.5 µM (for each of the lanes). Sonicated salmon sperm DNA at 0.5–1 µg/µl was added to the binding reactions to eliminate non-specific binding. In Langevin dynamics (panels A and C) the probability of bubble openings is represented by the same color map; red denotes high probability and blue denotes low probability of opening. The length of the transient bubbles, given in base pairs [bp], is shown along the vertical axis. The horizontal axis depicts base pair position; the bubble formation region is highlighted in blue while the points-of-contact are highlighted in yellow. The names of each of the sequences are shown in the panels while the complete nucleotide sequences could be found in Table 1.

We used EPBD Langevin dynamics to identify base pair substitutions that would have the effect of modifying FIS1 local breathing dynamics *without* modifying any direct points-of-contact. Our study resulted in design of a sequence that we will refer to as FIS1S (Table 1). This sequence differs from FIS1 as follows. In the regions flanking the 15 bp Fis core binding region, there are eight substitutions: at positions −13:A→C, −12:A→G, −11:A→C, −9:T→G, +9:A→C, +10:A→G, +11:T→C, and +13:T→G, that leave the direct points-of-contact with Fis intact. Moreover, in the core-binding region, but outside the bubble formation region, there are three substitutions: at position −6:T→C, −5:T→G, and +5:A→C. Each of these three substitutions has been shown individually not to affect Fis-DNA binding [51], [58]. EPBD simulations indicate that the above 11 substitutions together suppress the local DNA breathing dynamics (Figure 5A). Thus, based on our hypothesis concerning the relationship between breathing dynamics and affinity, we expect Fis to bind FIS1S more weakly than FIS1. To test this prediction, we performed EMSA experiments and found that binding of purified Fis protein to the FIS1S oligonucleotide is indeed weaker than binding to FIS1, *i.e.*, a higher concentration of protein is required to observe binding to FIS1S (Figure 5B). The FIS1 sequence (at 100 nM) forms a detectable complex with Fis protein at a concentration of 0.25–0.50 µM. In contrast, the FIS1S sequence forms a detectable complex only at a higher concentration of 0.75–1.0 µM (Figure 5B). Thus, the EMSA experiments agree with our prediction.

#### Strengthening Fis-DNA binding by enhancing breathing dynamics with O6-methylguanine modifications

To determine if we could enhance binding by modifying breathing dynamics, we used EPBD simulations to guide O6-methylguanine modifications of the FIS2 sequence, which binds Fis weakly. According to our hypothesis, modifications that enhance breathing dynamics, while leaving the direct points-of-contact with Fis intact, should strengthen Fis-DNA binding. We saw this effect by incorporating two O6-methylguanine (6mG) modifications (at position −2:G→6mG and +1:G→6mG) into the bubble formation region of the FIS2 sequence. We will refer to the modified sequence as FIS2^m2^ (Table 1). The O6-methylguanine modifications were chosen because the presence of a 6mG in a DNA segment destabilizes slightly the double helix and hence decreases its melting temperature [68] while causing only a minor perturbation of the Watson–Crick structure [69], [70], [71], [72]. A decrease in the melting temperature is an indicator of enhanced bubble dynamics at physiological temperatures and, indeed, the EPBD Langevin simulations show that FIS2^m2^ exhibits enhanced breathing (Figure 5C). Consistent with our expectations, the EMSA experiments demonstrate stronger binding of Fis to FIS2^m2^ than FIS2 (Figure 5D). In the case of FIS2^m2^, significant complex formation is observed at a Fis protein concentration of 0.25 µM, whereas there is no detectable FIS2 complex with Fis at this concentration. Binding of Fis to FIS2 can only be detected at protein concentrations ≥0.50 µM.

#### Weakening Fis-DNA binding by destabilization of the double helix with O6-methylguanine and mismatch modifications

**Figure 6:**
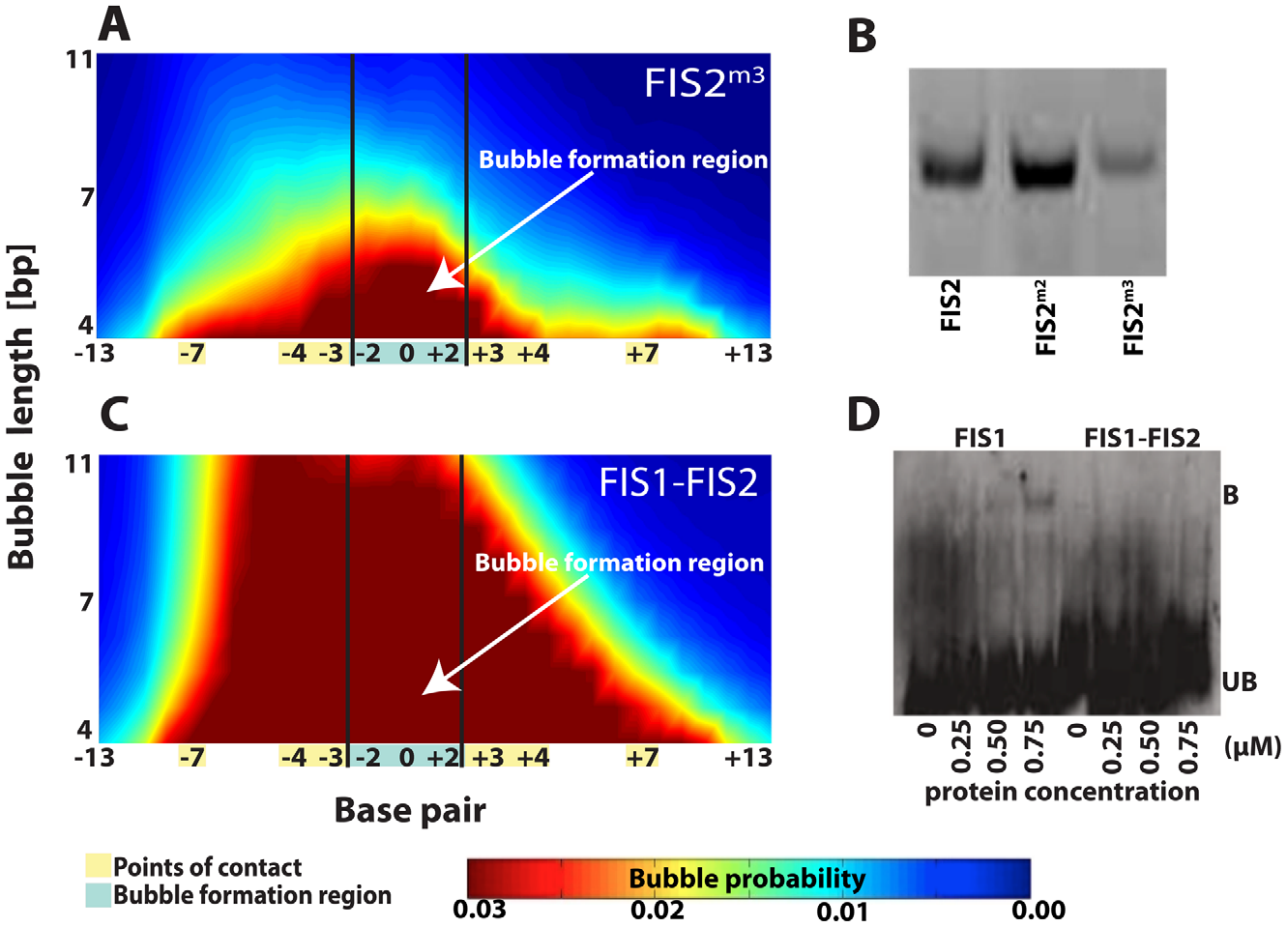
Fis binding site modifications, Langevin dynamics simulations of DNA breathing, and EMSA experiments, the second set. (A) Langevin dynamics simulations reinforcing the local DNA breathing dynamics in FIS2^m3^ via three O6-methylguanine modifications in the bubble formation region of FIS2 (Table 1). The direct points-of-contacts remain unchanged. (B) Polyacrylamide gel electrophoresis of dsDNA oligonucleotides sequences - FIS2, FIS2^m2^, and FIS2^m3^ - demonstrating gel migratory effects due to possible bubble formation (gel at 15%). (C) Langevin dynamics simulations demonstrating local disruption of the hydrogen bonds in the super-enhanced DNA local openings of the FIS1–FIS2 sequence (Table 1) caused by the presence of five mismatches at the FIS1 bubble formation region. (D) EMSA demonstrating the lack in complex formation in FIS1–FIS2. Concentration of the FIS1 and FIS1–FIS2 oligomers were constant at 100 nM and Fis protein ranged from 0 to 0.75 µM. Sonicated salmon sperm DNA at 0.5–1 µg/µl was added to the binding reactions to eliminate non-specific binding. In Langevin dynamics simulations (panels A and C) the probability of bubble openings is represented by the same color map as in Figure 5; red denotes high probability and blue denotes low probability of opening. The probability is determined from the lifetimes of all open states with a given length (bp) and above amplitude of 1.0 (Å), normalized over the complete time of the simulation. The length of the transient bubbles, given in base pairs [bp], is shown along the vertical axis. The horizontal axis depicts base pair position; the bubble formation region is highlighted in blue while the points-of-contact are highlighted in yellow. The names of each of the sequences are shown in the panels while the complete nucleotide sequences could be found in Table 1.

In contrast to, e.g., the *E. coli* SBB-protein, which can bind to a single DNA strand [73], Fis binds only to double-stranded regions but not to single stranded DNA. Therefore, we would expect the correlation between the enhanced breathing dynamics and Fis binding affinity to hold only below a threshold level of the propensity for transient openings of the double helix. To check if this is the case, we constructed another DNA sequence by introducing three O6-methylguanines into FIS2, which we will refer to as FIS2^m3^ (Table 1). FIS2^m3^ contains three 6mG modifications (at position −2:G→6mG, −1:G→6mG, and +1:G→6mG) that strongly destabilize the Fis binding site by locally disrupting the double helix. Indeed, Fis binds FIS2^m3^ weakly. The complex of Fis and FIS2^m3^ was barely detectable even at a Fis protein concentration of 1.0 µM (Figure 5D, the third set of lanes). Our EPBD simulations suggest that the bubble formation region of FIS2^m3^ has a much higher opening probability than other sequences considered so far (Figure 6A). In accordance with this enhanced local destabilization of the double helix structure, the FIS2^m3^ oligomer (alone) displays retardation in mobility compared to FIS2 and FIS2^m2^ oligomers (Figure 6B), potentially signifying that large bubble formation, disrupting locally DNA structure, can have slightly slow migration in the gel. This is an indication that the presence of extremely enhanced local transient openings in the bubble formation region is the likely reason for weak binding of Fis to the FIS2^m3^ sequence.

To further test this hypothesis, we designed a novel, FIS1–FIS2, hybrid sequence with five mismatches in the bubble formation region and no other modifications (Table 1). Our EPBD simulations indicate that FIS1–FIS2 is locally melted at the bubble formation region (Figure 6C). Accordingly, FIS1–FIS2 did not exhibit detectable binding by EMSA at Fis protein concentrations sufficient for Fis binding to FIS1 (*e.g.*, at a Fis concentration of 0.75 µM) (Figure 6D). These results support the conclusion that Fis binding requires an optimal level of DNA breathing, *i.e.*, that Fis binding is suppressed by both the absence of sufficient breathing and breathing above a threshold level.

In each of the performed experiments, multiple nucleotide modifications were introduced simultaneously. We recognize that this makes it difficult to separate the effect of these modifications on DNA breathing dynamics from their possible effects on other DNA physicochemical features that may impact Fis-DNA binding, such as flexibility. Nevertheless, the EPBD calculated breathing dynamic profiles accurately predict Fis-DNA binding strength for the examined sequences.

## Discussion

In this report, we studied the connection between DNA breathing dynamics and binding affinity of the Fis protein. In general, proteins bind to a particular DNA sequence by having a series of energetically favorable — electrostatic or van der Waals — contacts with the base pairs at the binding site. The specificity of the binding is believed to rely on two main recognition mechanisms [6], [74]: (i) direct recognition, which requires the presence of specific DNA nucleotides, the direct points-of-contact; and (ii) indirect recognition, which depends on the DNA conformation in the vicinity of the binding motif. Thus, DNA shape and entropic effects are important for indirect recognition. For example, protein binding to the major groove of DNA is perhaps primarily an enthalpy-driven process, based on the energetic profiles of protein–DNA structures, whereas binding to the minor groove is enabled by entropic effects [9]. It is natural to expect that the binding of a protein with direct points-of-contact *predominantly* on one of the two DNA strands, such as the TF and initiator YY1 [75], [76], can be promoted by transient openings of the DNA at the TF binding site, as it was demonstrated recently [47]. In contrast, a DNA sequence having low breathing propensity should be a better binding site for proteins that interact with both DNA strands, such as NF1 [77], [78], since an easily destabilized DNA motif (*i.e.*, one forming enhanced transient bubbles) would not be favorable for binding to both DNA strands [79]. Interestingly, initiator TFs mostly have points-of-contact at one of the DNA strands [80]. DNA breathing can expose nucleotides by base flipping, for example, for interaction with proteins, which could be favorable depending on the mechanism of protein-DNA binding. A binding mechanism that may benefit from breathing in this way could be similar to the mechanism proposed for methyltransferases [33]. Another connection between breathing and protein-DNA binding can be seen for proteins whose binding depends on local DNA bending [81], because of the interrelation between local bending and DNA breathing [82], [83], [84]. Although local DNA bending and propensity for breathing are interrelated, the propensity for breathing does not necessarily coincide with the bending-stiffness of a DNA sequence. For example, a poly(A) tract is internally unbent and stiffer than a general DNA sequence [85], but this tract is thermodynamically very soft and its melting temperature is one of the lowest [65], [86]. Thus, the interrelationship between breathing and bending is complex. DNA local static-curvature has also been associated with DNA binding by some nucleoid-associated proteins, including Fis [87], [88].

In the study reported here, we found that there is a strong correlation between *in vitro* Fis binding affinity and enhanced DNA breathing dynamics. Furthermore, we found that Fis binding sites in the *E. coli* genome are statistically associated with a characteristic breathing profile. Finally, in targeted experiments, we demonstrated that base pair substitutions in the flanking regions of the Fis binding motif that leave direct points-of-contact intact, as well as O6-methylguanine and mismatch modifications, can change DNA mechanics, with either enhancement or inhibition of DNA breathing and strengthening or weakening of *in vitro* Fis-DNA binding.

In conclusion, although the results of this study do not deconvolute the contribution of DNA breathing from other factors such as local DNA bending, or the sequence requirements of the inclusion/exclusion rules, our findings strongly suggest that Fis-DNA binding depends on a computationally derivable DNA breathing, and that using specific breathing profile as a feature for prediction of genomic Fis binding sites will be beneficial. It is noteworthy that DNA breathing depends on DNA mechanics, which can be impacted by genetic or other DNA modifications (e.g., mismatches or nucleotide methylation). Hence, TF binding that depends on DNA breathing could be affected by even a few such modifications in a nontrivial way. Future studies will be required to determine if the results reported here for Fis and in Ref. [47] for YY1 generalize to other TFs and to elucidate the molecular mechanisms by which transient DNA openings facilitate Fis-DNA binding.

## Materials and Methods

### Cloning and expression of GST fusions to response regulators

The Fis *E. coli* protein was amplified with sequence-specific primers: forward 5′-GATCGGATCCATGTTCGAACAACGCGTfAAATTCTGAC-3′ and reverse 5′-GATCAAGCTTTTAGTTCATGCCGTATTTTTTCAATTTTTTACGCAG-3′, containing *BamHI* and *HindIII* restriction enzyme sites, respectively, by PCR in 50 ml reactions [1 ml 100 mM primer 1, 1 ml 100 mM primer 2, 50 ng genomic DNA isolated from *E. coli* DH5α, 5 ml 10× Pfu reaction buffer, 1 ml 100 mM dNTPs, 2.5 ml DMSO, 2.5 U of PfuUltra DNA polymerase, and distilled H_2_O for the remaining volume] using the following conditions, (1) 94°C, 3 min, (2) 94°C, 1 min; 50°C, 1 min, 72°C, 1 min for 30 cycles, and (3) 94°C, 1 min; 50°C, 1 min, 72°C, 10 min. The Fis gene was cloned into the pGEX-KG vector using T4 ligase (NEB), transformed into BL21 *E. coli* competent cells, and induced for N-terminal GST fusion protein expression with 1 mM IPTG for 4 hrs. Cells were lysed with 1 mg/ml lysozyme on ice for 30 min, followed by treatment with 10 mg/ml DNase and 10 mM MgCl_2_ for an additional 30 min, and centrifuged at 40,000 rpm for 1 hr. GST fusion proteins were purified from the cleared supernatants by incubation with agarose beads cross-linked to glutathione for 1 hr and then eluted with 50 mM Tris-Cl (pH 8), 10 mM reduced glutathione. Protein samples were then dialyzed using a Slidealyzer cassette (Pierce) with a 10,000 MWCO to remove free glutathione, quantified using the BCA Protein Assay (Pierce), and stored at −80°C at a final concentration of 25% glycerol.

### Electrophoretic Mobility Shift Assays (EMSA)

Table 1 displays the oligonucleotide sequences used to demonstrate Fis binding to target DNA sequences. All dsDNA sequences used in EMSA experiments, including those with guanine modifications, were obtained through annealing of two synthetic oligonucleotides (Gene Link, Hawthorne, NY), in which all forward sequences were biotinylated at the 5′ end for detection. The annealing reaction was performed by incubating a 20 µM solution of the two oligonucleotides in dH_2_O at 95°C in a heat block for 5 minutes, removal of the block to the bench, and progressive decrease to room temperature to allow strand annealing to occur. The reaction mixtures consisted of 500 nM or 1.0 µM of dsDNA and protein concentrations of 250 nM–1.5 µM, in binding buffer [20 mM HEPES], 150 mM NaCl, 500 µg/mL BSA, 1 mM DTT, 0.1 mM EDTA, adapted from [51]. Sonicated salmon sperm DNA at 0.5–1 µg/µl was added to the binding reactions *to* eliminate non-specific binding. The reactions were allowed to incubate at room temperature for 30 minutes before loading onto a 6.0% non-denaturing polyacrylamide gel. The gel was pre-run for 30 minutes at 8 V/cm in 0.5× TBE buffer composed of 44 mM Tris-Cl, 44 mM Boric Acid, 1.0 mM EDTA pH 8.0, and samples were run at 17 mA for approximately 2.5 hours. Gels were developed using the LightShift Chemiluminescent EMSA kit (Thermoscientific) according to the manufacturer's instructions. Gels were transferred to nylon membrane (Thermoscientific) at 380 mA for 45 minutes. DNA was cross-linked to the membrane with 15 minutes exposure to ultraviolet light. Chemiluminescent detection was performed using a ChemiDoc XRS gel imaging system (BioRad). Oligonucleotide migration assays were conducted on 15% polyacrylamide gel at 120V for 3 hours. Concentration of oligonucleotides was 100 nM with 0.5× TBE buffer. Gels were stained with ethidium bromide and imaged with appropriate filters.

### Computer simulations

#### Extended Peyrard-Bishop-Dauxois (EPBD) model

To study DNA breathing dynamics, we used the mesocopic EPBD model, which is an extension of the original Peyrard-Bishop-Dauxois model [22] that includes sequence-specific stacking potentials [23].

A comment on the choice of model is perhaps appropriate, as many models have been used to study the mechanical properties of DNA. Most of them are purely thermodynamical models parameterized on the basis of measurements of equilibrium thermodynamical properties. The probabilities for local DNA opening obtained from the EPBD model are also equilibrium properties of the underlying free energy landscape, and essentially the same information can be obtained from various available thermodynamical models, such as the Poland-Sheraga model [25]. However, it should be noted that the EPBD model is a dynamical model that is strongly nonlinear and admits breather solutions, which constitute transient but relatively long-lived openings of the double helix, that are interconnected with the local bending propensity [84]. The EPBD derived trajectories contain the information about the lifetimes of the DNA transient openings (bubbles). This type of information cannot be obtained by purely thermodynamical calculations. Explicit accounting of the dependence on the solvent conditions such as salt, temperature, as well on the twist of the DNA, can lead to long-lived bubbles with enhanced lifetimes (see e.g., [89], [90]). Another advantage of the EPBD model is its single-nucleotide resolution. With a thermodynamical model, the calculation of a property profile typically requires window-averaging over 100–500 base pairs, which limits one's ability to distinguish the property profiles of two closely related sequences. In contrast, window averaging is not used in EPBD calculations, and as a result, the effects of even single base-pair changes can be readily determined.

The EPBD model is a quasi-two-dimensional nonlinear model that describes the transverse opening motion of the complementary strands of double-stranded DNA, while distinguishing the two sides (left - *v*_n_ and u right - *u*_n_) of the DNA double strand. The potential surface 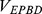 of the EPBD model is:
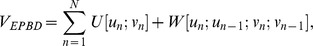
where
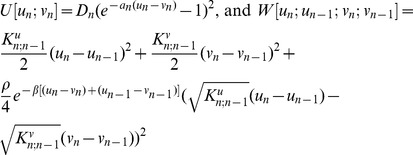
The sum is over all *N* base pairs of the DNA sequence. For each base pair, the EPBD includes two independent degree of freedom; *u*_n_ and *v_n_*, that represent the relative displacement from the equilibrium of the corresponding nucleotide, located in the right or left strand of DNA double helix. The displacements are chosen as in [22], and quantify the transverse stretching, 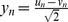 of the hydrogen bonds between the complementary nucleotides. The first term, 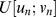, is the Morse potential for the n^th^ base pair. 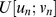 represents the combined effects of the hydrogen bonds between the complementary bases and electrostatic repulsion of the backbone phosphates [22]. The parameters *D_n_* and *a_n_* depend on the nature of the base pair (A-T *vs.* G-C, *i.e.*, two hydrogen bonds *vs.* three) at the base pair *n*. The second term 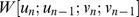 represents a quasi-harmonic approximation of the stacking interactions between consecutive nucleotides, which influences their transverse stretching motion. The exponential term effectively decreases the stacking interaction when one of the nucleotides is displaced away from its equilibrium position, *e.g.*, when one of the nucleotides is out of the DNA stack. The stacking force constants 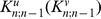 depend on the nature of the base, on its closest neighbor, and on the location of the nucleotide - the right or left DNA strand. The dinucleotide stacking force constants were determined in [23] by fitting simulation to UV-melting curves of DNA oligomers. The stacking force-constants are constructed in a manner that allows treatment of single strand DNA-defects such as UV-dimers [91], mismatches, and other defects that belong only to one of the DNA strand. In particular, this dependence is designed in a way such that the EPBD Hamiltonian to correspond to the PBD Hamiltonian, when the DNA sequence is homogenous. In the new variables the only changes of the parameters of the model are 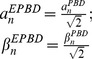. The mass and dispersion of the random force remains unchanged, while the value of any external force has to be divided by √2, in the corresponding Langevin stochastic differential equations for *u* ***_n_*** and *v_n_*.

#### EPBD based Langevin dynamics simulations

Langevin molecular dynamics simulations were performed at T = 310 K, by numerically integrating systems of stochastic differential equations corresponding to the EPBD model [38]. Periodic boundary conditions were applied to avoid terminal base pair effects without introducing torsional effects. Each DNA sequence was simulated in 1000 separate realizations, each with duration of 1 ns. At the flanking of each of the simulated sequences we added clamps, *viz.*, five base pairs (GCGCG) at the right side, and five base pairs (CGCGC) at the left side, to avoid end effects. The probability 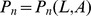 for the existence of a bubble of a certain length *L* [bp], beginning at base pair *n*, and with amplitude of the opening larger than *A* [Å], was calculated as in [38]: 
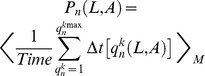
. Here, < >_M_ denotes averaging over all M = 1000 ensembles of stochastic trajectories, and *Time* is the total duration (1 ns) of each run, 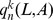 enumerates the bubbles of duration 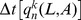 with amplitudes larger than *A* [Å] and length *L* [bp] beginning at the n^th^ base pair in the k^th^ simulation. Therefore, the probability is determined from the lifetimes of all open states with a given length *L* [bp] and above amplitude *A* [Å], normalized over the time of the simulation and over the ensemble of 1000 stochastic trajectories. In the simulations of mismatches (*i.e.*, the sequence FIS1–FIS2) the hydrogen bonds (*i.e.*, the force-constants *D_n_*) were set to zero at the locations of the mismatches, while the stacking force-constants 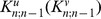 were chosen according to the type of the nucleotides at the right (*u_n_*) or left (*v_n_*) strand of the DNA.

#### EPBD based MCMC simulations and average displacement/opening profiles

For each base pair, the displacement from its equilibrium position quantifies the transverse stretching y_n_ of the hydrogen bonds between the complementary nucleotides (*i.e.*, the base pairs opening) as a function of DNA sequence. To obtain the average displacement/opening profile, for a specific DNA sequence at a given temperature, we performed Markov chain Monte Carlo (MCMC) simulations based on the EPBD model as described earlier [23], [39]. In each run, we first produced an equilibrium state using the standard Metropolis algorithm [92] and then recorded the displacements y_n_ of each base at selected time steps. Performing a large number of simulation runs (each with different initial conditions), we obtained the average displacement/opening profile (<y_n_>) of the DNA sequence of interest. Thus, <y_n_> represents the “thermodynamic softness” and “thickness” of the double helix because of the base pair breathing at physiological temperature and pH [23], [39], [46], [65]. The profile <y_n_> is related to DNA melting [62], [86], [93]. Indeed, DNA denaturation is a closed-to-open state transition of the double helix and the transition is quantified by measuring/calculating the fraction of disrupted hydrogen bonds (openings) between complimentary nucleotides (*i.e.*, the fraction of these base pairs, for which <y_n_> is bigger then a given threshold-distance) as a function of temperature. Importantly, the profile <y_n_> is free of the requirement for window averaging, usually applied in thermodynamic calculations, making the average displacement/opening profile sensitive to single base pair substitutions [39]. The profile <y_n_> can be calculated efficiently via MCMC simulation, and the results have been shown to be equivalent to those obtained by averaging over Langevin dynamics trajectories [39]. In the supporting information, we provide a listing of the parameter values of the EPBD model (Text S1), more information about the algorithms used in calculations, and links to sources of available software implementations of these algorithms.

#### EPBD based MCMC protocol for O6-methylguanine (6mG) modifications

Several powerful mutagens and carcinogens attack DNA at the O6-position of guanine [94], which result in O6-methylguanine. This guanine modification leads to a destabilization of the double helix and a base-flipping rate similar to the behavior of mismatched DNA segments [95]. Here, we use 6mG modifications to destabilize the DNA double helix and to enhance the local bubble formation at the Fis-DNA binding site. To derive the changes (due to the presence of 6mG) in the hydrogen bonds between the complementary nucleotides and in the stacking interactions with the closest consecutive nucleotides, we followed our protocol for deriving the dinucleotide stacking constants described in [23]. In particular, we use MCMC simulations to reproduce the melting behavior of three 21 bp long DNA sequences, whose UV-melting were experimentally determined with and without the presence of a single 6mG (41). The simulated three sequences are: **S1**:GGTGGGCGCTGGAGGCGTGGG, with melting temperature T_m_ = 73.5+/−0.3; the methylated **S2**:GGTGGGCGCTG **^m^G** AGGCGTGGG, with T_m_ = 68.4+/−0.3; and **S3**:GGTGGGCGCT **^m^G** GAGGCGTGGG, with T_m_ = 65.6+/−0.4. We first simulated the melting behavior of sequence **S1**, which does not contain 6mG modification. To achieve the ∼5°C difference in the melting temperatures between sequences **S1** and **S2**, we changed gradually the strength of the hydrogen bond force constant D_n_, at the place of the 6mG modification, with a step of 0.0075 eV. Finally, we obtained the difference of ∼3°C between the sequence **S2** and **S3** (both containing one 6mG modification but at different location) by varying the strength of the stacking force constant K_n;n-1_, at the place of the 6mG modification, in steps of 0.0025 eV/Å^2^. In the MCMC, the steps were performed using a cutoff value of 25 Å and a strand separation threshold of y_n_  = 0.5 Å, above which the DNA was considered melted. At least 1000 simulations with different initial conditions were conducted. The D_n_ for 6mG was found to be 0.0345 eV, the TG dinucleotide-stacking step was changed to 0.0075 eV/Å^2^. These values were further used in the Langevin dynamics simulations of the FIS1–FIS2 sequence.

### Datasets and statistical analysis

#### Datasets

*E. coli* K12 MG1655 genome was downloaded from the KEGG database [96]. The genomic locations of the *E. coli* open reading frames were retrieved form the KEGG database while the locations of *E. coli* promoters were downloaded from RegulonDB [66]. The data for *in vitro* Fis binding sites is described in Table S1, while the *in vivo* identified genomic *E. coli* Fis binding sites were taken from [56], [57], [60], [66]. For the genomic Fis binding site dataset, any entries duplicated in a single data source, lacking genomic coordinates, having a binding site length less than 15 bp, or more than 50 bp were discarded. All *in vivo E. coli* Fis binding sites analyzed in this study are summarized in Tables S2 and S3. For the random set, 50 bp long sequences were randomly selected from the *E. coli* K12 MG1655 genome. The random set was constructed to have the same percentage of sequences in promoter (60%), intergenic (6%), and open reading frame (34%) regions as the one in the Fis binding dataset. Each random sequence was selected to not contain (or overlap) with any other random sequence.

The Fis protein structure was downloaded from the protein database [97] with PDB code 3IV5 as submitted by [51], and visualized using the VMD software [98].

#### Statistical analysis

A specific characteristic opening profile (COP) was derived for the Fis protein. The maximum correlation between the subsequences of each randomly selected region with the COP was statistically compared with the maximum correlation between the COP and subsequences of the regions containing Fis binding sites. Two-sample T^2^ and two-sample Kolmogorov-Smirnov tests were used for the statistical comparison. In addition, these two statistical tests were applied to the correlations to the COP distributions of *all* subsequences in both sets.

#### SVM model and comparison how other models (BioBayesNet and CRoSSeD) recognize the motifs corresponding to the characteristic breathing profile

A dataset containing positive and ten times more negative examples was constructed. The positive examples, *i.e.*, ones corresponding to genomic Fis binding sites, were obtained by selecting the subsequences with maximum correlation with the COP profile in each of the experimentally determined Fis binding sites in [56], [57], [60], [66]. As before, any Fis binding sites duplicated in a single data source, lacking genomic coordinates, having a binding site length less than 15 bp or more than 50 bp were discarded. The negative examples were generated by randomly selecting 15 bp long sequences in a manner similar to the one described in the previous section.

Our SVM classifier was trained on the sequences' average breathing profiles, obtained by EPBD MCMC simulations, and by leveraging the LIBSVM framework and utilities [99]. The CRoSSeD model was trained using its default parameters after converting the sequences from FASTA format to the appropriate format using the CRoSSeD converter [14]. The BioBayesNet model [67] was trained with default settings – all structural parameters were used and no information for motifs or significant regions was provided to the tool. Each of the models was evaluated using five 10-fold cross-validations. Any bias from randomly splitting the dataset was avoided by repeating the 10-fold cross-validation five times with different splitting of the subsets. All reported results are averaged over the five 10-fold cross-validations.

## Supporting Information

Table S1 **Previously characterized** ***in vitro*** **Fis binding sites.** Information for all previously *in vitro* characterized Fis bindings site used in our analysis. Nucleotides located in bubble formation regions are highlighted in blue, while points-of-contact with DNA are highlighted in yellow.
(XLSX)

Table S2 **Summary of previous studies identifying Fis binding sites in the** ***E. coli*** **genome.**
(XLSX)

Table S3 **Examined Fis binding sites' locations in the** ***E. coli*** **genome.**
(XLSX)

Table S4 **Parameters of the EPBD model.**
(PDF)

Text S1 **EPBD model algorithms and implementations.** This supporting text provides a listing of the parameter values of the EPBD model, information about the algorithms used in our calculations, and links to source codes of available software implementations of these algorithms.
(PDF)
