# Supplementary material for: Binding of Nucleoid-Associated Protein Fis to DNA Is Regulated by DNA Breathing Dynamics
Source: PLoS Comput Biol. 2013 Jan 17;9(1):e1002881. doi: 10.1371/journal.pcbi.1002881 (PMC3547798; doi:10.1371/journal.pcbi.1002881)
Supplement: Table S4 — Parameters of the EPBD model. (PDF) [file pcbi.1002881.s004.pdf]

Table S4

The parameters of the EPBD model

Stacking Potential (EPBD) [a] $K^{b}_{n;n-1}$ [eV/Å²]		Stacking Potential (PBD)[c]	Morse Potential (PBD)[c]
K_{CG}=0.0280<br>K_{CA}=0.0250<br>K_{TG}=0.0250<br>K_{GC}=0.0249<br>K_{AT}=0.0230<br>K_{AG}=0.0229<br>K_{CT}=0.0229<br>K_{AA}=0.0228	K_{CG}=0.0280<br>K_{CA}=0.0250<br>K_{TG}=0.0250<br>K_{GC}=0.0249<br>K_{AT}=0.0230<br>K_{AG}=0.0229<br>K_{CT}=0.0229<br>K_{AA}=0.0228	β =0.35 /√2 [1/Å²]<br>ρ =2	D_{AT}= 0.05 [eV]<br>a_{AT} = 4.2/√2 [1/Å]<br><br>D_{GC} = 0.075 [eV]<br>a_{GC} = 6.9 /√2 [1/Å]

[a]The EPBD constants are derived in [1].

[b]The index u/v in the $K^{u}_{n;n-1}$ and $K^{v}_{n;n-1}$ is not shown because the force constants are the same for the neighbors located at the same strand. The index u/v is only useful in the case of presence of "defects" (e.g., for description of mismatches), note that the physically meaningful variable is only the relative displacement: $y_n=(u_n-v_n)/\sqrt{2}$.

[c]The PBD constants which remained the same in the EPBD are derived in [2]. Other constants are as follows: the mass **m**=0.0301 [a.u.], the friction in the preheating phase γ =0.05 [ps^{-1}], and the friction in the monitoring phase γ =0.005 [ps^{-1}] [3,4].

References

1. Alexandrov BS, Gelev V, Monisova Y, Alexandrov LB, Bishop AR, et al. (2009) A nonlinear dynamic model of DNA with a sequence-dependent stacking term. Nucleic acids research 37: 2405-2410.
2. Campa A, Giansanti A (1998) Experimental tests of the Peyrard-Bishop model applied to the melting of very short DNA chains. Phys Rev E 58: 4.
3. Alexandrov BS, Gelev V, Yoo SW, Bishop AR, Rasmussen KO, et al. (2009) Toward a detailed description of the thermally induced dynamics of the core promoter. PLoS computational biology 5: e1000313.
4. Alexandrov BS, Wille LT, Rasmussen KO, Bishop AR, Blagoev KB (2006) Bubble statistics and dynamics in double-stranded DNA. Physical review E, Statistical, nonlinear, and soft matter physics 74: 050901.
