# Supplementary material for: Binding of Nucleoid-Associated Protein Fis to DNA Is Regulated by DNA Breathing Dynamics
Source: PLoS Comput Biol. 2013 Jan 17;9(1):e1002881. doi: 10.1371/journal.pcbi.1002881 (PMC3547798; doi:10.1371/journal.pcbi.1002881)
Supplement: Text S1 — EPBD model algorithms and implementations. This supporting text provides a listing of the parameter values of the EPBD model, information about the algorithms used in our calculations, and links to source codes of available software implementations of these algorithms. (PDF) [file pcbi.1002881.s005.pdf]

Text S1

1. Monte Carlo Markov chain (MCMC) protocol

Our **MCMC** protocol is based on the standard Metropolis-Hastings algorithm [1].
MCMC source code (written in C) is available at this URL:
http://prl.aps.org/supplemental/PRL/v102/i2/e029602.
The length of the monitoring phase is 150,000 MC steps, and the number of runs (with different initial conditions) is 1000. If the software cited above is used, the input sequence should be given in the **L42B18.dat** file before running the program. The parameters of the stacking and Morse potentials used in our calculations are given in **Table S4**. Note that these parameters are used in the place of a single constant k. The **MCMC** protocol has been described previously [2].

2. Langevin Dynamics (LD)

Our **LD** protocol is based on the standard (for Langevin Dynamics) generalized stochastic Verlet algorithm [3].
LD source code (written in FORTRAN) is freely available at this URL:
http://www.ccl.net/cca/software/SOURCES/FORTRAN/allen-tildesley-book/f.33.shtml
If the software cited above is used, the potential should be substituted with the EPBD potential (see **Table S4**) and the hydrodynamic tensor set to a constant. A detailed description of the Langevin equation with inertia as well as Langevin Dynamics is available elsewhere [4] .
In our **LD** simulations the monitoring phase is 50,000,000 steps, and the number of runs (with different initial conditions) is 1000. The parameters of the stacking and the Morse potentials are given in **Table S4**. Our **LD** protocol has been described previously [5,6].

References

1. Metropolis N, Rosenbluth AW, Rosenbluth MN, Teller AH, Teller E (1953) Equations of state calculations by fast computing machines. J Chem Phys 21: 6.
2. Alexandrov BS, Gelev V, Monisova Y, Alexandrov LB, Bishop AR, et al. (2009) A nonlinear dynamic model of DNA with a sequence-dependent stacking term. Nucleic Acids Research 37: 2405-2410.
3. Schlick T (2010) Molecular Modeling and Simulation: An Interdisciplinary Guide, Second Edition: Springer.
4. Allen MP, Tildesley DJ (1989) Computer Simulation of Liquids: Oxford University Press, USA.
5. Alexandrov BS, Gelev V, Yoo SW, Bishop AR, Rasmussen KO, et al. (2009) Toward a detailed description of the thermally induced dynamics of the core promoter. PLoS computational biology 5: e1000313.
6. Alexandrov BS, Wille LT, Rasmussen KO, Bishop AR, Blagoev KB (2006) Bubble statistics and dynamics in double-stranded DNA. Physical review E, Statistical, nonlinear, and soft matter physics 74: 050901.
